# Parental physical activity, parental mental health, children’s physical activity, and children’s mental health

**DOI:** 10.3389/fpsyt.2024.1405783

**Published:** 2024-06-19

**Authors:** Gavin Davidson, Lisa Bunting, Claire McCartan, Anne Grant, Orla McBride, Ciaran Mulholland, Emma Nolan, Dirk Schubotz, Julie Cameron, Mark Shevlin

**Affiliations:** ^1^ School of Social Sciences, Education and Social Work, Queen’s University Belfast, Belfast, United Kingdom; ^2^ Regional Trauma Network, Northern Health and Social Care Trust, Holywell Hospital, Antrim, United Kingdom; ^3^ School of Nursing and Midwifery, Queen’s University Belfast, Belfast, United Kingdom; ^4^ School of Psychology, Ulster University, Coleraine, United Kingdom; ^5^ School of Medicine, Dentistry and Biomedical Sciences, Queen’s University Belfast, Belfast, United Kingdom; ^6^ Department of Psychiatry and Behavioural Neurosciences, McMaster University, Hamilton, ON, Canada; ^7^ Mental Health Foundation, McLellan Works, Glasgow, United Kingdom

**Keywords:** parental physical activity, parental mental health, children’s physical activity, child mental health, family focused interventions

## Abstract

**Introduction:**

The benefits of physical activity for mental health and well-being and the associations between parental mental health and children’s mental health have been well established. These important issues tend to be examined separately however, and there is limited research on the associations between parent and child physical activity and mental health when all considered together. While family focused practice is recommended to provide support for parents who have mental health problems and their families and includes various components (such as psychoeducation, support for mental health and parenting), promoting physical activity for parents and children is not usually a core component of these interventions.

**Methods:**

The Northern Ireland Youth Wellbeing Survey aimed to provide estimates of the prevalence of mental health problems among children and young people. The survey also included questions about parental physical activity, parental mental health, and children’s physical activity (for those aged 11–19 years). The main aim of the analysis reported in this article was to explore possible bivariate associations between parent and child physical activity and mental health and also explore these associations when all considered together. Participants were included in the analysis where there were completed interviews for the young person and one of their parents, and both young person and parent provided responses in relation to questions on weekly physical activity (*n* = 882).

**Results:**

The findings highlight the positive associations between parental physical activity and parental mental health, and between children’s physical activity and children’s mental health. They also explore some of the more complex interactions between these four variables, which suggest that gender may also be an important consideration. There were significant associations between father’s physical activity and son’s mental health, and son’s physical activity and father’s mental health.

**Discussions:**

These findings suggest that including support for parental physical activity and children’s physical activity should be a routine component of family focused mental health interventions. It is important to acknowledge that there may be additional barriers to engaging in physical activity for families where a parent is experiencing mental health problems, and these should also be explored and addressed.

## 1 Introduction

The associations between physical activity and mental health have been repeatedly confirmed, and highlighted in relation to promoting mental well-being and also responding to mental health problems. Perhaps most prominently, the New Economics Foundation’s *Five ways to wellbeing* (1) identified evidence-based approaches to promoting well-being, which included to *be active* (along with *connect*, *take notice*, *keep learning*, and *give*). There is also increasing recognition of the reduced life expectancy of people with mental health problems. Chan et al. (2) conducted a systematic review and meta-analysis and reported that, based on 109 studies, the pooled years of potential life lost was 14.66 years. In Northern Ireland, McCarter et al. (3) linked hospital data on the main life limiting conditions, from 2010 to 2021, with diagnoses of severe mental illness. They reported that, after adjusting for other variables, those with a diagnosis of severe mental illness had a twofold excess likelihood of mortality. Although there are a range of factors associated with the mortality gap in mental illness, higher rates of sedentary behavior and low levels of physical activity are significant contributors (4). Sedentary behavior and low physical activity levels are also associated with a range of other lifestyle risk behaviors that impact physical and mental well-being and increase the risk of cardiovascular disease including poor diet, smoking and alcohol misuse (5). Much of this evidence includes data from parents but there is some, although more limited, evidence of the association specifically between parental physical activity and parental mental health (6, 7).

Mahindru et al. (8) reviewed the possible mechanisms for the associations between physical activity and mental health, and these include complex physiological, psychological, and contextual processes. It should also be acknowledged that, for people experiencing mental health problems, there may be additional barriers to engaging in physical activity (9). Encouragingly, physical activity has been found to be an effective intervention for adults’ (10, 11) and children’s mental health problems (12, 13).

There is also a substantial body of research on the associations between parental mental health problems and children’s mental health problems (14, 15). Leijdesdorff et al. (16) reported that the 15%–23% of children who live with a parent with a mental illness have an up to 50% risk of developing a mental illness. Risks are elevated as a result of a complex interplay between a range of processes including the impact of the illness on parenting, increased family conflict, and challenging socio-economic circumstances (17). Similarly, as parent and child mental health is associated, levels of physical activity in parents are often reflected in those of their children. Petersen et al. (18) included 39 articles on the association between parent and child physical activity and reported a positive relationship across studies, which was similar across the gender of parent–child dyads. Neshteruk et al. (19) have highlighted that research on children’s physical activity has focused on the role of mothers and that more research is needed on the role of fathers. Sigmundova et al. (20) used pedometers to measure physical activity in parent–child dyads. They found that, with younger children, aged 4–7 years, the mother–daughter association was the strongest and with children aged 8–16 years, it was the father–son relationship. The current physical activity guidelines, from the National Health Service (21) in the UK, recommends that children and young people should be aiming for an average of at least 60 min of moderate or vigorous intensity physical activity a day across the week and that adults should be doing at least 150 min of moderate intensity activity or 75 min of vigorous activity spread evenly over four to 7 days a week (22).

A less commonly explored aspect of the literature is the possible complex multi-layered interactions between physical activity and mental health in families. Based on a large-scale survey of parents (*N* = 10,141) in four South American countries during the COVID-19 pandemic, Ben Brik et al. (6) found that parents who reported more frequent physical activity also tended to report lower anxiety for them and their child. Sutcliffe et al. (23) explored the associations between having a child involved in organized sport and parental mental health using data from a longitudinal study in Australia. Highlighting the complexity of the issues involved, they reported that parents with adolescents involved in organized sport reported higher levels of life stress and time pressure but lower levels of psychological distress.

Interventions designed to support families where a parent has mental health problems tend to neglect the promotion of physical activity despite the extant knowledge of its effectiveness for many mental health problems. Family-focused interventions often include a number of core components: psychoeducation, direct treatment and support for mental health and/or substance use, parenting behavior, child risk and resilience, family communication, family support and functioning, and access to community supports and services (24). Promoting physical activity for parents and children is rarely a core component of these interventions despite recommendations that physical health issues should be a greater focus of mental health interventions (25).

## 2 Materials and methods

### 2.1 Research design and aim

The Northern Ireland Youth Wellbeing Survey (NIYWS) not only aimed to provide estimates of the prevalence of mental health problems among children and young people but also included some data about parents. A more detailed account of the rationale and methods for the survey is also available (26). This survey created the opportunity to explore the possible associations between parental physical activity, parental mental health, children’s physical activity, and children’s mental health. With four variables, there are six possible pairs of relationships. Bivariate correlations were therefore used to examine the relationships between parent physical activity and their own mental health, young person physical activity and their own mental health, parent physical activity and their child’s physical activity, parent mental health and their child’s mental health, parent’s physical activity and their child’s mental health, and the young person physical activity and their parent’s mental health. To account for the non-independence of these parent and child dyads, we used the Actor–Partner Interdependence Model (APIM) (27) to investigate the associations between adolescent and parent physical activity on their own mental health (“actor effects”) and with the mental health of the other member of the dyad (“partner effects”). This statistical approach acknowledges the potential interdependence of findings from people in close relationships such as parents and their children (28).

### 2.2 Sample

The NIYWS recruited a random probability sample, stratified by deprivation decile and by the six counties of Northern Ireland to ensure a representative sample. Addresses were selected from a dataset of addresses. Participants were a representative sample of the 2- to 19-year-old population of NI. Only young people, aged 11–19 years, and parents were asked about physical activity. For this study, a sub-set of participants were therefore included, where both the young person and one of their parents had completed interviews and both had provided responses in relation to the question on weekly physical activity (*n* = 882).

### 2.3 Measures

#### 2.3.1 Mental health

Adolescent mental health problems were measured using the Revised Children’s Anxiety and Depression Scale (RCADS; 29). The RCADS is a 47-item questionnaire that produces indications of clinically relevant levels of severity of six disorders derived from the diagnostic criteria of the DSM-IV (30): major depressive disorder, separation anxiety disorder, social phobia, generalized anxiety disorder, panic disorder, and obsessive compulsive disorder. One of the more widely used brief screening instruments for symptoms of anxiety and depression, RCADS has shown robust internal consistency reliability in different assessment settings, countries, and languages (31), good test–retest reliability (29), and good convergent validity (32). Importantly, it has shown good reliability and validity within a population of Irish youth aged 12–18 years (33). The scale is available in formats that can be self-completed or completed by a parent/carer; the parent version has been validated for use with children aged 3–17 years (34). In this study, 11- to 19-year-olds completed the self-report version. Each item is scored on a 4-point Likert response scale (0 = *never* to 3 = *almost always*), and raw subscale scores are converted into *t*-scores, which are normed based on school year and gender. This process is facilitated using syntax available from the developer that identifies cutoff scores above the clinical threshold (https://www.childfirst.ucla.edu/resources). The dichotomized rate for a young person meeting “clinical” threshold for any of these common mood and anxiety disorders is used in this analysis.

Current possible mental health problems among parents were assessed using the General Health Questionnaire (GHQ-12; 35). The GHQ-12 is a widely used screening measure for identifying possible mental health problems in the general population and has been used in the Northern Ireland Health Survey (36), Understanding Society survey (37) and the Adult Psychiatric Morbidity Survey (38). It is a 12-item self-completion questionnaire, which yields a maximum score of 12, with a score of 4 or more typically used to identify individuals with mental health problems.

#### 2.3.2 Physical activity

Parents and young people, aged 11–19 years, were asked “In a typical week, how many days do you do moderate to vigorous physical activity?” Answers ranged from 0 to 7 with responses indicating that their level of activity “was too varied to say” scored as missing.

#### 2.3.3 Gender

Parent and child age and gender (male, female, other) were self-reported.

### 2.4 Data collection

The data were collected between 1 June 2019 and 19 March 2020, and so data collection was completed just before the COVID-19 restrictions. After an initial approach by letter, experienced interviewers visited the selected addresses and used computer-assisted personal interviewing to collect the data.

### 2.5 Data analysis

The data were analyzed using SPSS V29 and Jamovi V2.4.11. Descriptive statistics were produced for all study variables. Bivariate correlations were then used to examine the relationships between parent mental health, young person mental health and levels of parent and young person physical activity. To account for the non-independence of parent and adolescent dyads, we used the APIM (27) to investigate the associations between adolescent and parent physical activity on their own mental health (“actor effects”) and with the mental health of the other member of the dyad (“partner effects”). Structural equation modelling (SEM) was implemented using Jamovi software. Parent and young person physical activity variables were simultaneously entered as independent (exogenous) variables, and the total score for parents and young people’s mental health symptoms was entered simultaneously as dependent variables (endogenous). All endogenous variables were simultaneously regressed on the exogenous variables and the residuals for the endogenous variables were correlated. Dyad members were treated as distinguishable, and the model included those who had complete data for both the parent’s and adolescent’s mental health and physical activity (882 dyads in total). Maximum likelihood estimation method was used to simultaneously estimate all the model parameters. The *R* ^2^ for each endogenous variable was used as an estimate of effect size. Follow-up analysis included running the same model with parent gender as a multi-group analysis factor and then dyad gender (mother/daughter, father/son, mother/son, father/daughter) with a subsample of young people who lived with both biological parents (*N* = 575).

### 2.6 Ethics

Ethical approval was granted by Queen’s University Belfast’s School of Sciences, Education and Social Work’s School Research Ethics Committee. As the survey was exploring potentially sensitive issues, there was a clear protocol to outline consent, anonymity, and confidentiality (and its limits), safeguarding and responding to distress.

## 3 Results

### 3.1 Descriptive statistics

Most of the parent participants were female (77.6%), and just over half of the young people were male (51.4%). **Table 1** presents descriptive statistics for the parents’ and young people’s mental health physical activity measures. Twenty-five percent of parents met the cutoff score of 4 or above on the GHQ-12 for likely mental health problems (*M* = 2.36, *SD* = 3.29), while 16% of young people met the cutoff for any mood or anxiety disorder on the RCADS. In terms of frequency of moderate to vigorous physical activity in a typical week, 15% of parents reported that they did not engage in any such physical activity, 47% reported that they did so between 1 and 3 days per week, and 38% said they did so between 4 and 7 days a week. For the young people, 5% reported 0 days a week, 47% reported 1–3 days per week, and 48% reported moderate to vigorous physical activity on 4–7 days a week. Continuous scores for these variables were used in subsequent analysis.

**Table 1:** Frequencies of parent and adolescent mental health and physical activity measures and gender.

	Parent		Young person	
Mental health problems	*N*	%	*N*	%
Yes	223	25.3	139	15.9
No	659	74.7	735	84.1
Physical activity				
None	133	15.1	46	5.2
Low (1–3 days)	413	46.8	414	46.9
High (4–7 days)	336	38.1	422	47.8
Gender				
Male	197	22.4	453	51.4
Female	684	77.6	429	48.6

### 3.2 Correlations

Bivariate correlations examining the relationships between parent mental health, young person mental health, and levels of parent and young person physical activity are presented in **Table 2**. These showed small but significant positive associations between parent and young person mental health and small but significant negative associations between both parent mental health and parent physical activity and young person mental health and physical activity. They also show a small but significant positive association between parent and young person physical activity and a small but significant negative association between young person physical activity and parent mental health but not for young person mental health and parent physical activity. The mean score for parents’ mental health, as measured by the GHQ, was 2.23 (*SD* = 3.23), and for young people’s mental health, as measured by the RCADS, was 30.93 (*SD* = 23.84). The mean for parents’ weekly physical activity was 3.05 days (*SD* = 2.10) and for young people it was 3.48 days (*SD* = 1.94).

**Table 2:** Correlation matrix for parent and young people mental health and physical activity measures.

	Parent GHQ score	Young person RCADS score	Parent physical activity	Young person physical activity
**Parent GHQ score**	1			
**Young person RCADS score**	.146**	1		
**Parent physical activity**	−.212**	−0.066	1	
**Young person physical activity**	−.082*	−.200**	.143**	1
**Mean**	2.23	30.90	3.05	3.48
**SD**	3.23	23.84	2.10	1.94

**Correlation is significant at the 0.01 level (2-tailed) * Correlation is significant at the 0.05 level (2-tailed).

### 3.3 Actor–partner interdependence model

**Figure 1:**
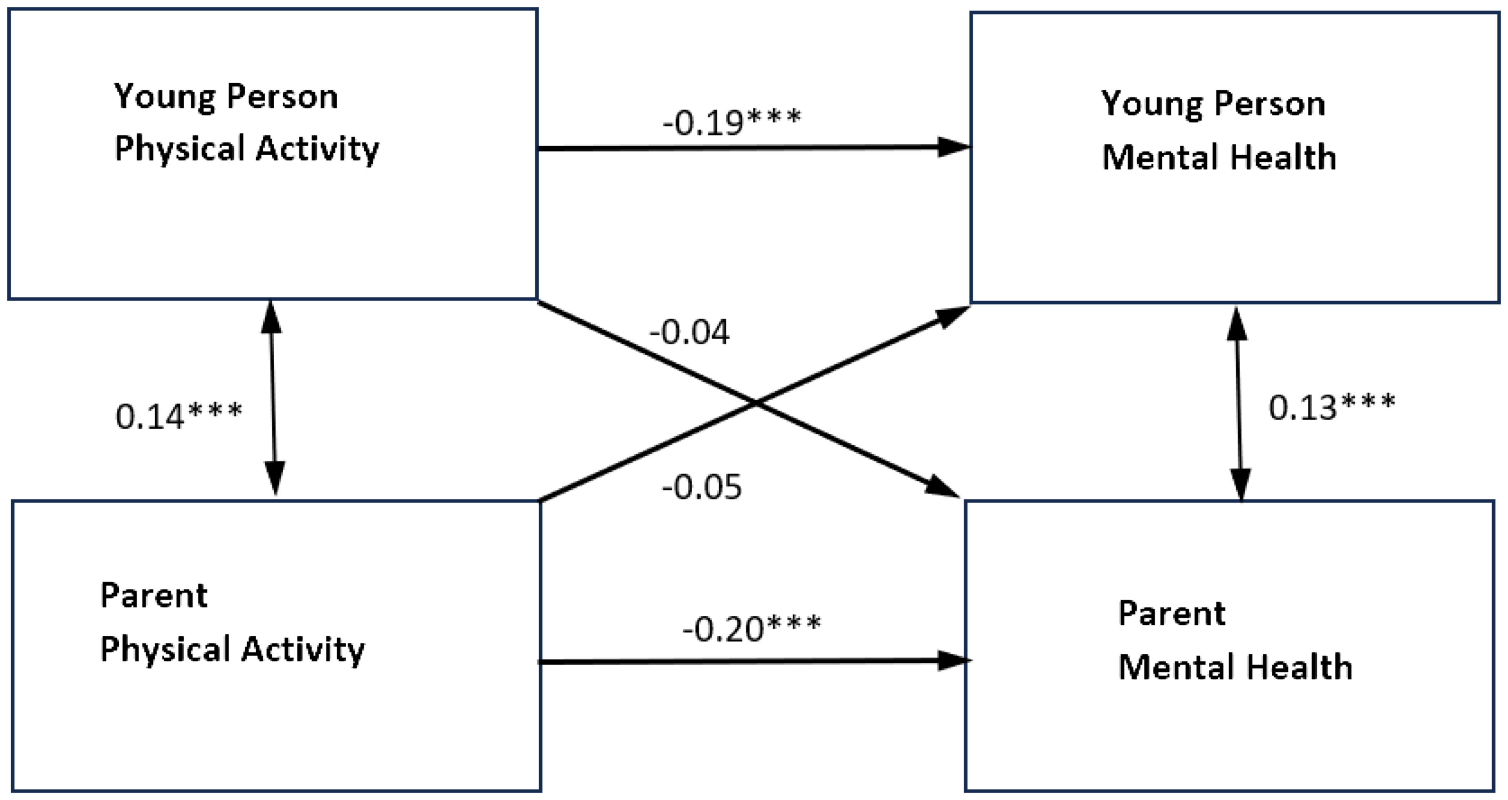
Standardized parameter estimates of actor and partner effects for parent and adolescent physical activity in relation to self-reported mental health symptoms. ***p<0.001 **p<0.01 *p<0.05. Single headed arrows represent the β values for the actor and partner effects. The double headed arrow between "Young Person Physical Activity" and "Parent Physical Activity" represents its covariance. The double headed arrow between "Parent Mental Health" and "Young Person Mental Health" is the residual nonindependence in these outcome scores, which is represented by the covariance between their corresponding two error terms.

Results from the APIM SEM model showed significant actor effects for both parents and young people (**Figure 1** and **Table 3**). The actor effect for parents was equal to −0.32 (*p* <.001, 95% CI [−0.42, −0.22]), with an overall standardized effect of −0.20. The actor effect for young people was equal to −2.35 (*p* <.001, 95% CI [−3.19, −1.59]) and the overall standardized actor effect was −0.19. The partner effects were non-significant for both groups. The *R* ^2^ was.048 for parental mental health and.042 for young people’s mental health, indicating a small effect size (4%–5% of the variance in endogenous variables being explained).

**Table 3:** Actor–partner interdependence model estimates for the relationship between physical activity and mental health by role of adolescent and parent (*N* = 882).

Person/role	Estimates	95% CI		β	95% CI		*p*
		Lower	Upper		Lower	Upper	
Young Person Physical Activity (Actor)	**−2.39**	**−3.19**	**−1.59**	**−0.19**	**−0.26**	**−0.13**	**< .001**
Parent Person Physical Activity (Partner)	**−**0.43	**−**1.18	0.31	**−**0.04	**−**0.10	0.03	0.25
Parent Person Physical Activity (Actor)	**−0.32**	**−0.42**	**−0.22**	**−0.20**	**−0.27**	**−0.14**	**< .001**
Young Person Physical Activity (Partner)	**−**0.09	**−**0.20	0.02	**−**0.05	**−**0.12	0.01	0.11

The rows in bold are statistically significant.

**Figure 2:**
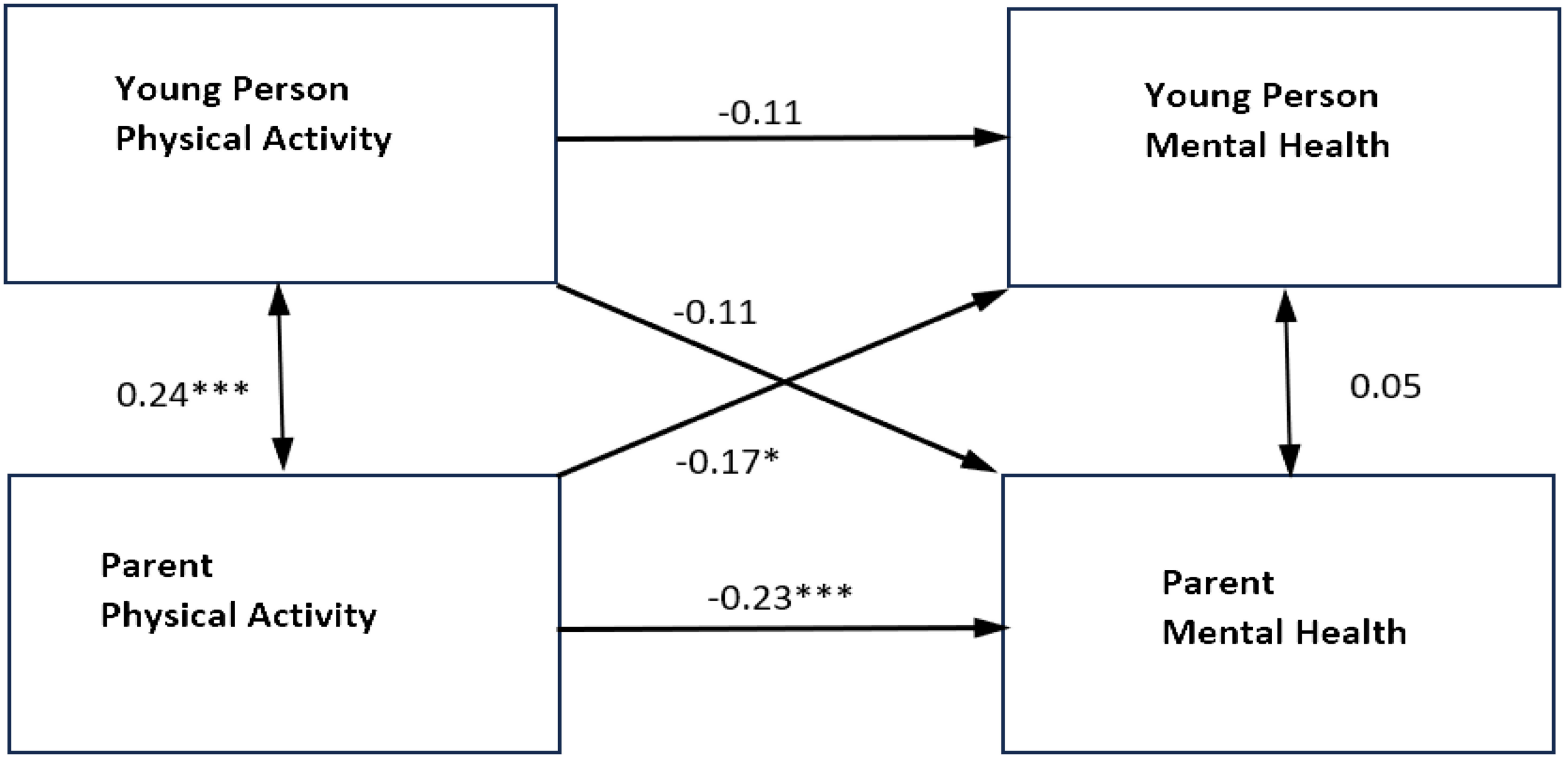
Standardized parameter estimates of actor and partner effects for fathers and adolescent physical activity in relation to self-reported mental health symptoms. ***p<0.001 **p<0.01 *p<0.05. Single headed arrows represent the β values for the actor and partner effects. The double headed arrow between "Young Person Physical Activity" and "Parent Physical Activity" represents its covariance. The double headed arrow between "Parent Mental Health" and "Young Person Mental Health" is the residual nonindependence in these outcome scores, which is represented by the covariance between their corresponding two error terms.

**Figure 3:**
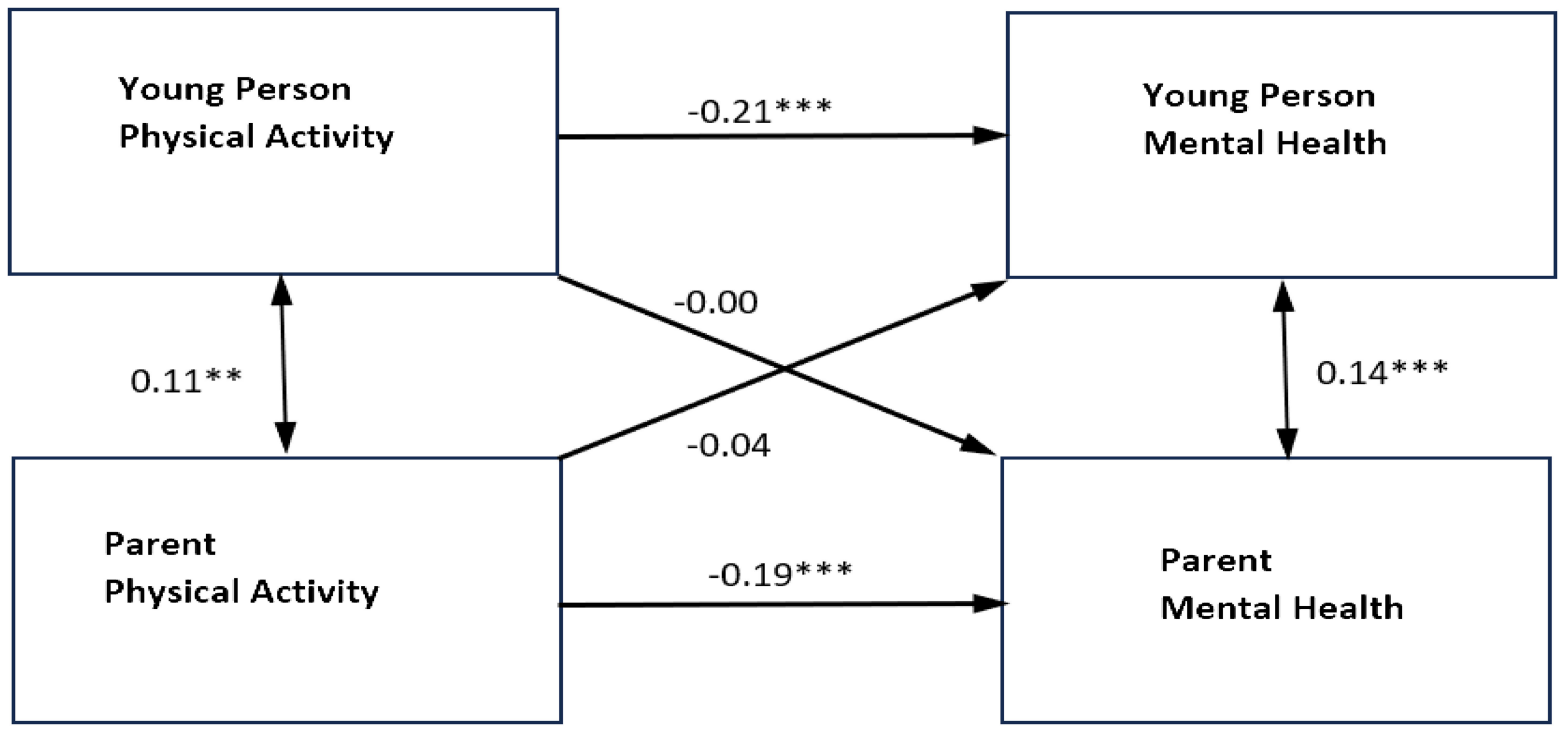
Standardized parameter estimates of actor and partner effects for mothers and adolescent physical activity in relation to self-reported mental health symptoms. ***p<0.001 **p<0.01 *p<0.05. Single headed arrows represent the β values for the actor and partner effects. The double headed arrow between "Young Person Physical Activity" and "Parent Physical Activity" represents its covariance. The double headed arrow between "Parent Mental Health" and "Young Person Mental Health" is the residual nonindependence in these outcome scores, which is represented by the covariance between their corresponding two error terms.

When the model was re-estimated for mothers and fathers separately, the results for mothers were similar with only significant actor effects for physical activity on the mental health of mothers and young people (for results, see **Figures 2**, **3**, and **Table 4**). The actor effect for the mothers was equal to **−**0.31 (*p* <.001, 95% CI [**−**0.43, **−**0.19]), with an overall standardized effect of **−**0.19. The actor effect for young people was equal to **−**2.63 (*p* <.001, 95% CI [**−**3.55, **−**1.71]) and the overall standardized effect was **−**0.21. The *R* ^2^ was 0.040 for mother’s mental health and 0.045 for young people’s mental health.

**Table 4:** Actor-partner interdependence model estimates for the relationship between physical activity and mental health by role of adolescent and parent and parent gender (N=881).

Person/Role	Estimates	95% CI		β	95% CI		*p*
		Lower	Upper		Lower	Upper	
Fathers							
Young Person Physical Activity (Actor)	−1.29	−2.89	0.31	−0.11	−0.25	0.03	0.115
Parent Person Physical Activity (Partner)	**−1.69**	**−3.09**	**−0.30**	**−0.17**	**−0.31**	**−0.03**	**0.017**
Parent Person Physical Activity (Actor)	**−0.31**	**−0.49**	**−0.12**	**−0.23**	**−0.36**	**−0.10**	**0.001**
Young Person Physical Activity (Partner)	−0.17	−0.38	0.04	−0.11	−0.25	0.03	0.113
Mothers							
Young Person Physical Activity (Actor)	**−2.63**	**−3.55**	**−1.71**	**−0.21**	**−0.28**	**−0.14**	**< .001**
Parent Person Physical Activity (Partner)	−0.03	−0.90	0.84	0.00	−0.08	0.07	0.938
Parent Person Physical Activity (Actor)	**−0.31**	**−0.43**	**−0.19**	**−0.19**	**−0.26**	**−0.12**	**< .001**
Young Person Physical Activity (Partner)	−0.07	−0.20	0.06	−0.04	−0.12	0.03	0.272

The rows in bold are statistically significant.

In the fathers’ model, there were significant actor effects for fathers’ physical activity on their own mental health, as well as significant partner effects for fathers’ physical activity on the young person’s mental health. The actor effect for the fathers was equal to **−**0.31 (*p* = 0.001, 95% CI [**−**0.49, **−**0.12]), with an overall standardized effect of **−**0.23. The partner effect of father’s physical activity on the young person’s mental health was equal to -1.69 (*p* = 0.017, 95% CI [**−**3.09, **−**0.30]), with an overall standardized effect of **−**0.17. This indicated that, as fathers’ levels of physical activity increased, young people’s mental health symptoms decreased. There were no significant actor effects for young people physical activity in relation to their own mental health or partner effects in relation to their father’s mental health The *R* ^2^ was 0.078 for fathers’ mental health and 0.051 for young people’s mental health.

Further analysis was conducted using the sub-sample of young people living with both biological parents (*N* = 575), to explore the effect of different gender groupings within family relationships (mother/daughter, father/son etc.) [**Table 5**]. While the smaller sample size and increased number of groups reduced many of the effects to marginal significance, a number of interesting patterns emerged.

**Table 5:** Actor–partner interdependence model estimates for the relationship between physical activity and mental health by parent–adolescent gender groups (*N* = 575).

Group/role	Estimates	95% CI		β	95% CI		*p*
		Lower	Upper		Lower	Upper	
Father–son							
Young Person Physical Activity (Actor)	0.12	−1.82	2.07	0.01	−0.20	0.22	0.902
Parent Person Physical Activity (Partner)	−**1.83**	−**3.49**	−**0.17**	−**0.23**	−**0.44**	−**0.03**	**0.03**
Parent Person Physical Activity (Actor)	−0.14	−0.40	0.11	−0.12	−0.33	0.09	0.27
Young Person Physical Activity (Partner)	−**0.29**	−**0.60**	**0.01**	−**0.21**	−**0.41**	**0.00**	**0.055**
Father–daughter							
Young Person Physical Activity (Actor)	−0.22	−3.90	3.45	−0.01	−0.26	0.23	0.905
Parent Person Physical Activity (Partner)	−1.89	−4.72	0.93	−0.16	−0.40	0.08	0.188
Parent Person Physical Activity (Actor)	−0.22	−0.51	0.08	−0.18	−0.41	0.06	0.147
Young Person Physical Activity (Partner)	−0.17	−0.56	0.21	−0.11	−0.34	0.13	0.384
Mother–son							
Young Person Physical Activity (Actor)	−**1.22**	−**2.54**	**0.11**	−**0.13**	−**0.26**	**0.01**	**0.072**
Parent Person Physical Activity (Partner)	0.40	−0.84	1.65	0.04	−0.09	0.18	0.526
Parent Person Physical Activity (Actor)	−**0.21**	−**0.43**	**0.02**	−**0.13**	−**0.26**	**0.01**	**0.071**
Young Person Physical Activity (Partner)	−0.13	−0.37	0.11	−0.07	−0.21	0.06	0.295
Mother–daughter							
Young Person Physical Activity (Actor)	−**2.88**	−**4.65**	−**1.11**	−**0.21**	−**0.34**	−**0.09**	**0.001**
Parent Person Physical Activity (Partner)	0.20	−1.46	1.87	0.02	−0.11	0.15	0.81
Parent Person Physical Activity (Actor)	−**0.30**	−**0.51**	−**0.10**	−**0.19**	−**0.32**	−**0.07**	**0.004**
Young Person Physical Activity (Partner)	−0.03	−0.25	0.19	−0.02	−0.15	0.11	0.796

The rows in bold are statistically significant.

**Figure 4:**
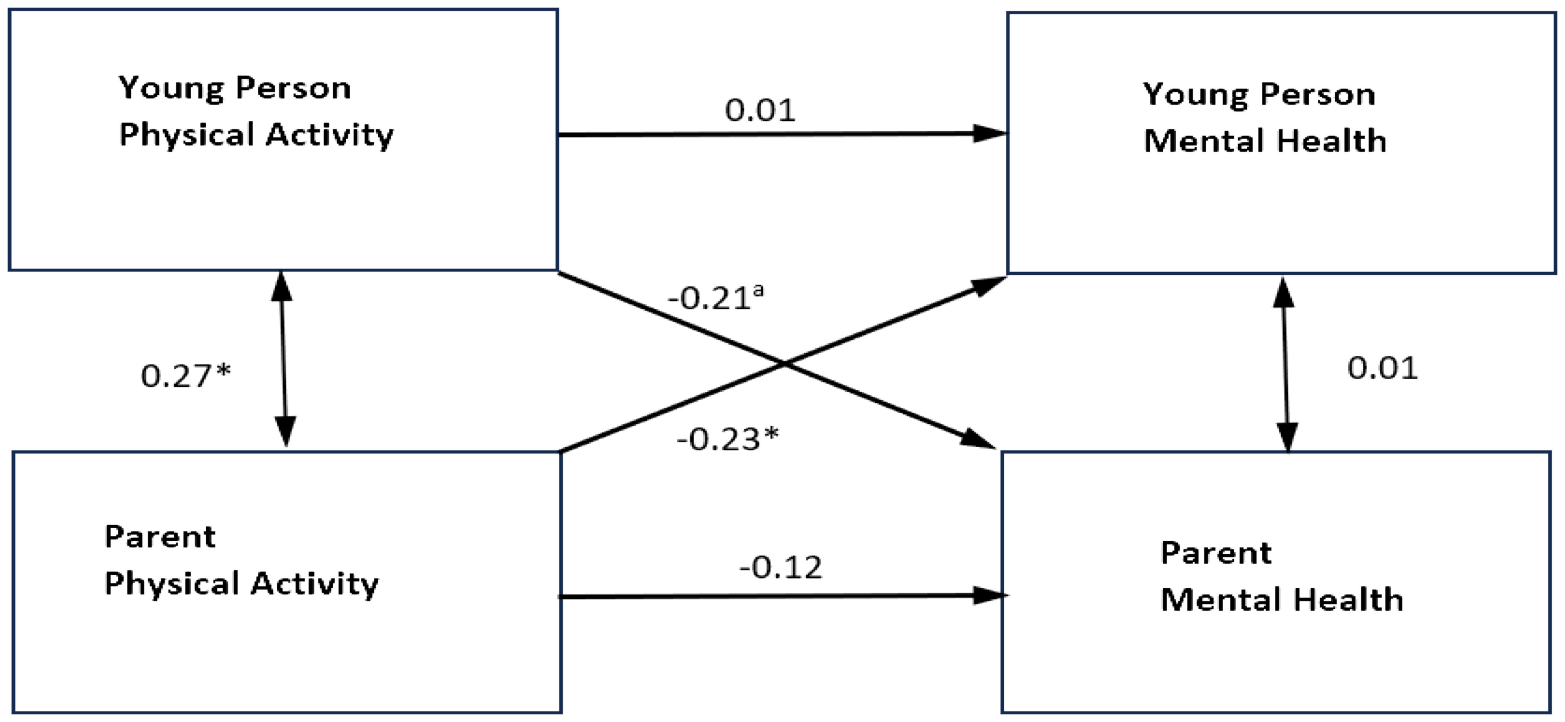
Standardized parameter estimates of actor and partner effects for father and son physical activity in relation to self-reported mental health symptoms. ***p<0.001 **p<0.01 *p<0.05. Single headed arrows represent the β values for the actor and partner effects. The double headed arrow between"Young Person Physical Activity" and "Parent Physical Activity" represents its covariance. The double headed arrow between "Parent Mental Health" and "Young Person Mental Health" is the residual nonindependence in these outcome scores, which is represented by the covariance between their corresponding two error terms.

For father–son dyads (**Figure 4**), there were no significant actor effects but there were significant partner effects of father’s physical activity on their son’s mental health (**−**1.83, *p* = 0.03, 95% CI [**−**3.49, **−**0.17]), and son’s physical activity on their father’s mental health (**−**0.29, *p* = 0.055, 95% CI [**−**0.60, 0.01]). The standardized partner effects were similar for both fathers (β = **−**0.20) and sons (β = **−**0.21), although the partner effect of son’s physical activity on their father’s mental health was only marginally significant. The *R* ^2^ in the father–son group was 0.069 for fathers’ mental health and 0.053 for sons’ mental health.

For both mother–daughter and mother–son dyads, there were significant actor effects, but no significant partner effects. In mother–son dyads the actor effect was **−**0.21 (*p* = 0.071, 95% CI [**−**0.43, 0.02]) for mothers and **−**1.22 (*p* = 0.072, 95% CI [**−**2.54, 0.11]) for sons. In mother–daughter dyads the actor effect was **−**0.30 (*p* = 0.004, 95% CI [**−**0.51, **−**0.10]) for mothers and **−**2.88 (*p* = 0.001, 95% CI [**−**4.65, **−**1.11]) for daughters. The standardized actor effects were similar for both mothers (β = **−**0.20) and daughters (β = **−**0.22), and for mothers (β = **−**0.13) and sons (β = **−**0.13), although the effect in the mother–son group was only marginally significant. In the mother–son group, the *R* ^2^ was 0.023 for mothers’ mental health and 0.017 for sons’ mental health, and in the mother–daughter group, it was 0.039 for mothers’ mental health and 0.045 for daughters’ mental health.

There were no actor or partner effects for father–daughter dyads, and *R* ^2^ was 0.051 for fathers’ mental health and 0.028 for daughters’ mental health.

## 4 Discussion

The findings from the bivariate correlations reinforce the wider literature on the positive associations generally between physical activity and mental health with small but significant associations between parent physical activity and parent mental health, young person physical activity and young person mental health. They also suggest the need to consider these issues in the family context, as the results of this study found positive associations between parent physical activity and young person physical activity, parent mental health and young person mental health, and parent mental health and young person physical activity. The only variables, which were not significantly associated, were parent physical activity and young person mental health.

The APIM analysis was used as it can explore the relationship between parent and young person physical activity and mental health. It acknowledges the likely mutual influence in these close relationships and looks at the within and between person effects. The actor effects: parent physical activity and parent mental health and young person physical activity and young person mental health, remained significant. This is perhaps the clearest and most important finding. It does suggest that generally mental health interventions should more routinely include a focus on physical activity.

The APIM analysis, when not split by gender, did not reveal any significant partner effects between parent physical activity and young person mental health, or between young person physical activity and parent mental health. However, when split by parents’ gender, there was a significant partner effect between fathers’ physical activity and young person mental health. Further analysis, which included young person gender, found that for the mother–daughter and mother–son dyads the actor effects were significant and the partner effects were not, in line with the overall parent and young person analysis. For the father–daughter dyads, there were no actor or partner effects and, for the father–son dyads, there were no significant actor effects but there was significant partner effects (father’s physical activity and son’s mental health, and son’s physical activity and father’s mental health) were significant. This further analysis suggests that gender may be an important consideration and that there may be something different about the specific relationships between fathers and their children when exploring physical activity and mental health.

An important limitation of these findings is that they are from a cross-sectional survey and so any discussion of possible direct or indirect causal relationships between variables has to be tentative. The underrepresentation of fathers in the sample is another potential limitation but also suggests the need to consider how to further promote the inclusion of fathers in family focused research, and supports Neshteruk et al. (19) suggestion that further research on the influence of the fathers is necessary, and potentially critical in understanding these associations. A further, specific limitation was that the single question about physical activity, although informed by the current guidelines for physical activity, did not provide detailed data. This could be addressed in future research by including more detailed assessment of the level, frequency and types of physical activity.

Overall these findings reinforce the need to further explore and promote the relationship between physical activity and mental health including in the family context and particularly when families have parents with mental health problems. The findings also suggest that the role of gender may be important to explore further, including in the design of interventions. Promotion of physical activity has not tended to be identified as a key component of family focused practice in the context of parental mental health problems, or of the training of the professionals involved, but these findings suggest that it should be. Existing reviews of research on interventions to promote physical activity in all families provide some helpful guidance about how this may be done although they also highlight more research is needed (39–42). In addition to facilitating access to, or directly providing interventions designed to promote physical activity in all families, there are opportunities to include this important aspect of support in the main, traditional components of interventions for families where a parent has mental health problems. The importance of physical activity and its benefits could be included in psychoeducation, including acknowledgement and exploration of possible barriers. Physical activity interventions could be integrated into direct treatment and support for mental health and/or substance use. Interventions focused on parenting and family functioning could also promote physical activity. Support for accessing wider community services could also include identifying opportunities for physical activity. These findings therefore have implications for the training of mental health professionals as well as for the design and delivery of interventions and services.

## Data availability statement

The datasets presented in this article are not readily available because access is restricted for a specified period as agreed with the funder. Requests to access the datasets should be directed to g.davidson@qub.ac.uk.

## Ethics statement

This study was approved by Queen’s University Belfast’s School of Sciences, Education and Social Work’s School Research Ethics Committee. The study was conducted in accordance with the relevant legislative and institutional requirements. Written informed consent for participation in this study was provided by the participants or participants’ legal guardians/next of kin.

## Author contributions

GD: Writing – original draft. LB: Writing – original draft. CM: Writing – original draft. AG: Writing – review & editing. OM: Writing – review & editing. CM: Writing – review & editing. EN: Writing – review & editing. DS: Writing – review & editing. JC: Writing – review & editing. MS: Writing – original draft.
